# Comparison of the Shear Bond Strength of Silorane-Based Composite Resin and Methacrylate Based Composite Resin to MTA

**DOI:** 10.15171/joddd.2018.001

**Published:** 2018-03-14

**Authors:** Buse Ayse Serin, Muharrem Cem Dogan, Hamdi Oguz Yoldas

**Affiliations:** ^1^Department of pediatric dentistry, Faculty of Dentistry, University of Cukurova Adana-Turkey; ^2^Department of Restorative dentistry, Faculty of Dentistry, University of Cukurova.Adana-Turkey; ^3^Department of Endodontics, Faculty of Dentistry, Cukurova University, Adana, Turkey

**Keywords:** Mineral trioxide aggregate, silorane-based composite resin, shear bond strength

## Abstract

***Background.*** Mineral trioxide aggregate (MTA) is a material that has recently gained popularity in the application of the vital pulp therapy. Along with the increasing use of MTA to this end, the permanent restoration material to be placed on MTA has become a significant issue. The aim of this in vitro study was to investigate the bond strength of the novel low-shrinkage silorane-based composite resin (SBC) to MTA.

***Methods.*** Twenty acrylic blocks filled with MTA were prepared for this study. SBC was the test group and methacrylate-based composite resin (MBC) was used as the control group. Shear bond strength test was performed to determine the bond strength. The surfaces of broken samples were evaluated under a stereomicroscope and grouped as adhesive, cohesive and mixed. Data were examined by statistical analysis.

***Results.*** Statistical analysis revealed that SBC exhibited higher shear bond strength than the control group. It was observed that most of the failures in the test group were of cohesive type within MTA.

***Conclusion.*** Based on the results, SBC showed higher shear bond strength than the control group; however, clinical follow-up is needed to evaluate the clinical success.

## Introduction

Mineral trioxide aggregate (MTA) has been regarded as an ideal material for perforation repair, retrograde filling, pulp capping and apexification since its introduction in 1993. MTA is a combination of a kind of mineral powder which consists of tricalcium sulfate, bismuth oxide, dicalcium silicate, tricalcium aluminate and calcium sulfate dihydrate. There are two types of MTA, namely gray and white, and both formulations are identical except that the gray MTA contains tetracalcium aluminoferrite. In the presence of moisture, MTA sets into a hard mass by hydration, becomes a colloidal gel similar to calcium hydroxide and reaches a pH of 12.5. Several studies have demonstrated its excellent sealing ability and biocompatibility.^1^

MTA is recommended as an alternative pulp capping agent for vital pulp therapy in pediatric dentistry.^2^ While the use of MTA in vital pulp therapy has gained popularity, what to place over MTA as a permanent restorative material has become a crucial issue. However, the adhesion of restorative materials to MTA has not been studied extensively and thus it is not very well-known.

Introduced in 2007, silorane-based composite resin is a low-shrinkage posterior restorative composite resin. This composite resin can only be used by its own self-etch adhesive system.^3^ Silorane is obtained from the reaction of oxirane and siloxane molecules. SBC exhibits low polymerization shrinkage due to the ring-opening oxirane monomer and increases hydrophobicity due to the presence of siloxane species.^3^

This in vitro study aimed to compare the shear bond strength of silorane-based composite resin and methacrylate-based composite resin to MTA.

## Materials and Methods

**Figure 1:**
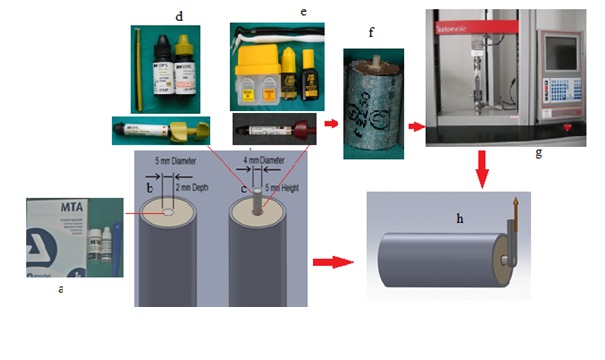
Schematicrepresentation of the ‘materials and methods’.* a:* White MTA (Angelus, Brasil). *b:* Stainless steel cylindrical molds were prepared in appropriate sizes (4 cm in length and 2.7 cm in diameter) as universal testing devices and these molds were filled with acrylic resin. A central hole, measuring 2 mm in height and 5 mm in diameter, was prepared on the cylindrical acrylic blocks. The holes were filled with MTA. *c:* Test and control group materials were applied on the MTA surface with a cylindrical-shaped plastic matrix with a diameter of 4 mm and a height of 5 mm. *d:* Test group material; Filtek Silorane System Adhesive, a self-etch, bond and composite resin, 3M ESPE, USA.* e:* Control group material; Clearfil SE Bond primer, bond, Kuraray Medical Inc., Okayama, Japan and Filtek Z250, 3M ESPE, St. Paul, MN, USA. *f:* The photograph of a sample ready for testing. *g:* Testometric Ax, M500-25kN, Rochdale, England. *h:* Shear bond strength test.

Twenty specimens of MTA (Angelus, Brasil) were prepared by using cylindrical acrylic blocks. For specimens of the test and control groups, stainless steel cylindrical molds were prepared in appropriate sizes (4 cm in length, 2.7 cm in diameter) as universal testing devices (Testometric Ax, M500-25kN, Rochdale, England) and these molds were filled with acrylic resin. A central hole, measuring 2 mm in height and 5 mm in diameter, was prepared on the cylindrical acrylic blocks. Standardization of the hole was achieved using a caliper and a periodontal probe. The schematic representation of the ‘materials and methods’ is presented in Figure 1.

The holes were filled with MTA which was mixed in line with the manufacturer’s instructions and covered with a wet cotton pellet and temporary filling material (Cavit, ESPE America Inc., Norristown, PA, USA). Then, the specimens were stored at 37°C under 100% humidity for 24 hours to create the necessary setting. After the removal of the temporary material, the MTA surface was not polished.

The blocks were randomly divided into two groups. The MTA surface was dried for 10 seconds to provide a dry surface.

For the test group, the self-etch primer (Filtek Silorane System Adhesive, Self-etch, 3M ESPE, USA) was applied to the surface for 15 seconds with a black microbrush, air-dispersed carefully and light-cured for 10 seconds. The adhesive bond (Filtek Silorane System Adhesive, self etch, 3M ESPE, USA) was then applied similarly with a green microbrush and light-cured for 10 seconds. SBC (Filtek Silorane Low-shrinkage Posterior Restorative, 3M ESPE, USA) in A2 shade was applied incrementally into cylindrical shaped plastic matrix with a diameter of 4 mm and a height of 5 mm by means of incremental technique; each increment was light-cured with a light-emitting diode light-curing unit (Elipar Freelight 3M ESPE) for 40 seconds.

For the control group, self-etch primer (Clearfil SE Bond primer, Kuraray Medical Inc., Okayama, Japan) was applied for 20 seconds and dried thoroughly with a mild air stream. Then the adhesive bond (Clearfil SE Bond Bond, Kuraray Medical Inc., Okayama, Japan) was applied, air-dried gently and light-cured for 10 seconds. MBC (Filtek Z250, 3M ESPE, St. Paul, MN, USA) in A2 shade was applied into a cylindrical plastic matrix with a diameter of 4 mm and a height of 5 mm by means of an incremental technique and then light-cured with a light-emitting diode light-curing unit (Elipar Freelight, 3M ESPE) for 40 seconds.

The polymerized specimens were stored in distilled water at room temperature for 24 hours. The specimens were inserted into the slot with the help of a screw, fixed and then sheared with a ring blade in a universal test machine (Testometric Ax, M500-25kN, Rochdale, England) at a crosshead speed of 1.0 mm/min. The time of fracture was recorded in Newtons of force, next the shear bond strength was calculated in megapascals (MPa) by dividing the peak load at failure by the specimen surface area.

Fractured surface micromorphology was examined under a stereomicroscope after the test at a magnification of ×25. Fracture modes were classified as follows: cohesive failure within MTA, cohesive failure within composite, adhesive failure that occurred at the MTA‒composite resin interface or mixed failure when the two modes of failure happened.

### Statistical Analysis

The mean shear bond strengths of specimens were compared by post hoc Tukey tests. Statistical analyses were carried out using SPSS 16.0 at P=0.09.

## Results

In this study, the bond strength of MTA to SBC was evaluated. The results were compared with a conventional MBC. The shear bond strength test was performed to evaluate the bond strength.

The means and standard deviations of shear bond strength values between MTA and SBC and MBC are presented in Table 1. Applying SBC with its self-etch adhesive system on MTA showed the highest mean shear bond strength of 11.1**±**4.7 MPa while using MBC with a conventional self-etch adhesive system on MTA showed a mean shear bond strength of 10.5**±**3.5 MPa. Significant differences were found between the groups.

**Table 1:** Descriptive statistics of shear bond strengths for each group

**Group**	**N**	**Mean ± SD (MPa)**
**MTA + SBC**	10	11.1**±**4.7
**MTA + MBC**	10	10.5**±**3.5

The mean differences of the groups were presented in Table 2. The shear bond strength of the test group (MTA + SBC) was significantly higher than that of the control group (MTA + MBC) (P=0.09).

**Table 2:** Mean differences of the groups were compared with post hoc Tukey tests

**Group**	**Group**	**Mean difference**	**Significance**
**MTA + SBC**	**MTA + MBC**	.61000	.009^*^
**MTA + MBC**	**MTA + SBC**	-.61000	.009^*^

* The mean difference is significant at the 0.05 level.

Table 3 shows the fracture modes of the groups. For the test group (MTA + SBC), most of the cohesive failures were observed in MTA. Adhesive failures were associated with lower bond strength values. Cohesive failure within composite was not observed in any group. Mix failures were observed mostly in the control group.

**Table 3:** Fracture modes of the groups

**Group**	**N**	**Adhesive**	**Mix**	**Cohesive**
**MTA + SBC**	**10**	**0**	**3**	**7**
**MTA + MBC**	**10**	**3**	**5**	**2**

## Discussion

MTA was first described in the dental literature in 1993.^4^ In 1998, the use of MTA for clinical purposes was permitted by FDA. When used in the vital pulp treatment, MTA has certain advantages such as hard tissue formation by stimulating the release of cytokines in the osseous cells, dentinogenic effect on the pulp, antimicrobial properties, noncytotoxicity, and ensuring the vitality of the pulp after pulp capping and pulpotomy.^5^ Based on these advantages in vital pulp treatment, MTA has gained significant recognition recently. With the increasing use of MTA, however, the kind of permanent restoration material to be placed on it and the bond strength between the permanent restorative material and MTA have become critical issues to consider. However, very few studies are available regarding the bond strength of MTA and restorative materials in the dental literature.^6-8^ These studies have evaluated the bond strength of MTA and methacrylate-based composite resin, glass-ionomer cement, compomers and different self-etch adhesive systems.

The most important reasons of failure in conventional methacrylate-based composite resin restorations are polymerization shrinkage which might lead to microleakage, tooth deformation, enamel cracks, secondary caries and postoperative sensitivity.^9^ Numerous studies have been performed in order to reduce polymerization shrinkage of composite resins, including modifying the content of resin and different application techniques.^10-19^

The low-shrinkage silorane-based composite resin is a new class of resin compound used in dentistry. The polymerization process of this composite resin occurs via a cationic ring-opening reaction which results in a lower polymerization contraction, compared to that of the methacrylate-based composite resin which polymerizes via a radical addition reaction of their double bonds. The combination of the two chemical building blocks of siloxanes and oxiranes provides the biocompatible, hydrophobic and low-shrinkage silorane-based composite restorative system.

Bond strength tests are used to evaluate the clinical performance of restorative and adhesive systems.^20-22^ Shear, tensile, microshear or microtensile tests are used to evaluate the bond strength of a composite resin.^23-30^ Shear bond strength test was preferred in this study since it is a more reliable and practical method.^31-33^

Higher shear bond strength values of ‘MTA + SBC’ than that ​​of ‘MTA + MBC’, which were used as control group, were found in this study. The lower shear bond strength of the control group, Clearfil SE bond and Filtek Z250, might be due to the inadequate effect of self-etch adhesive system. Clearfil SE Bond was used in the control group; the primer of this self-etch system consists of MDP (10-methacryloxydecyl dihydrogenphosphate). As already known, the acidic effect of MDP is brought about by interaction with water.^34^ Thus, when MTA was hardening, due to water use, MDP could not exert enough of its acidic effect.^35^ In addition to insufficient effect of the self-etch system used in the control group, MTA’s indented surface might have increased the micromechanical retention of low-shrinkage silorane-based composite resin.

The fracture surfaces were evaluated following the test. In the test group (MTA+SBC), the majority of the fractures were cohesive failures in MTA. Cohesive fractures have exhibited increased shear bond strength in the literature.^36-38^ The cohesive fracture in MTA was confirmed by the results. The adhesive fracture did not occur in the test group.

Although less polymerization shrinkage is clinically advantageous, it raises some questions. Reducing shrinkage stress deteriorates the mechanical properties of composite resins, decreasing the degree of polymerization. When the degree of polymerization declines, the amount of the residual monomer that does not participate in the reactions increases. These residual monomers have adverse effects on the bond strength. In an ideal composite resin, low polymerization shrinkage is expected when creating a high polymerization degree. Accordingly, some studies evaluating the bond strength of silorane-based composite resin to teeth have shown that the amount of silorane-based composite resin was lower than the amount of conventional methacrylate-based composite resin.^39-41^

An overall evaluation of the findings of this study revealed that silorane-based composite resin should be placed on MTA in the vital pulp therapy. However, due to the unsatisfactory results of studies assessing the performance of silorane-based composite resins and a lack of clinical studies, in vivo studies are needed before clinical use is adopted.

## Acknowledgments

None.

## Competing interests

The authors declare no competing interests with regards to the authorship and/or publication of this article.

## Ethics approval

Not applicable.
